# The impact of infection with COVID-19 on the respiratory microbiome: A narrative review

**DOI:** 10.1080/21505594.2022.2090071

**Published:** 2022-06-28

**Authors:** Taiping Zhu, Jun Jin, Minhua Chen, Yingjun Chen

**Affiliations:** a Internal Medicine Department, Chun’an Maternal and Child Health Hospital, Hangzhou, Zhejiang, China; b Emergency and Critical Care Center, Intensive Care Unit, Zhejiang Provincial People’s Hospital (Affiliated People’s Hospital Hangzhou Medical College), Hangzhou, Zhejiang, China; c Department of Infectious Diseases, Tiantai People’s Hospital of Zhejiang Province (Tiantai Branch of Zhejiang People’s Hospital), Taizhou, Zhejiang, China

**Keywords:** COVID-19, microbiome, respiratory microbiome, microbiome dysbiosis, immune responses

## Abstract

Coronavirus disease 2019 (COVID-19), caused by SARS-CoV-2, has affected millions of individuals with various implications. Consistent with the crucial role of the microbiome in determining health and disease in humans, various studies have investigated the gut and respiratory microbiome effect on the COVID-19. Microbiota dysbiosis might support the entry, replication, and establishment of SARS-CoV-2 infection by modulating various mechanisms. One of the main mechanisms that the modulation of respiratory microbiota composition during the COVID-19 infection affects the magnitude of the disease is changes in innate and acquired immune responses, including inflammatory markers and cytokines and B- and *T*-cells. The diversity of respiratory microbiota in COVID-19 patients is controversial; some studies reported low microbial diversity, while others found high diversity, suggesting the role of respiratory microbiota in this disease. Modulating microbiota diversity and profile by supplementations and nutrients can be applied prophylactic and therapeutic in combating COVID-19. Here, we discussed the lung microbiome dysbiosis during various lung diseases and its interaction with immune cells, focusing on COVID-19.

## Introduction

Although severe acute respiratory syndrome coronavirus-2 (SARS-CoV-2) was first reported in China’s Hubei province in November 2019, it rapidly spread globally and caused Coronavirus Disease 2019 (COVID-19) pandemic disease [1]. Despite the approval of some preventive vaccines and the availability of vaccinization programs, COVID-19 has affected around 311 million people worldwide by now (11 January 2022). Mild fever, fatigue, dry cough, sore throat, diarrhoea, and anosmia are the most common symptoms in SARS-CoV-2-infected patients [2]. Many infected people remain asymptomatic or have only mild upper respiratory tract symptoms, but others exhibit acute respiratory distress syndrome (ARDS) as well as pneumonia, necessitating intubation that can experience fatal complications [3]. Alongside respiratory complications, infection with SARS-CoV-2 can cause acute kidney injury, vascular thrombosis, endothelial sloughing, and shock [4]. Thus, multi-organ failures and complications imposition great stress for healthcare systems and prolonged hospitalization [5].

The microbiome is an aggregate of microorganisms, including bacteria, fungi, viruses, and protozoans, that reside in a particular microenvironment on or within the human body and are involved actively in cellular metabolisms and functions [6]. There is evidence that the interaction between microbes and humans plays a decisive role in determining the health or disease status in the human body owing to the contribution of microbiomes to the improvement or impairment of immune and metabolic functions [7]. Thus, understanding host-microbe interactions and dysbiosis of the microbiome could be useful in diagnosing and introducing appropriate treatment for diseases. Due to the high expression of the angiotensin-converting enzyme 2 (ACE2), a receptor of the SARS-CoV-2 virus, in respiratory and gastrointestinal tracts [8], some emerging scientific and clinical investigations have indicated the dysbiosis of the gut and respiratory microbiome during COVID-19 infection [9,10]. For example, the analyses of stool microbiome from 15 patients during hospitalization in Hong Kong revealed that COVID-19 patients exhibited remarkably reduced bacterial diversity and increased abundance of opportunistic ones, such as *Rothia*, *Streptococcus*, *Actinomyces*, *and Veillonella* [11]. On the other hand, the invasion of the SARS-CoV-2 virus towards the lungs for beginning COVID-19 disease is related to the lung infection and subsequent immune responses in which the lung microbiome might play an essential role in the initiation, development, and response to therapeutic agents. This review will summarize the lung microbiome dysbiosis during various lung diseases and its interaction with immune cells and responses. We focused on the alterations in the lung microbiome in patients infected with COVID-19 and how these alterations can be used to improve treatment outcomes.

## Lung microbiome dysbiosis during diseases

The studies based on the traditional culture systems have reported that the bronchial tree in healthy individuals is sterile [12,13]. Nowadays, the advent of high-throughput sequencing technologies and culture-independent techniques, such as sequencing the 16S rRNA gene, revealed that the bronchial tree is not sterile, even in healthy people. The lungs are a microbial ecosystem with a collection of microorganisms, both viable and non-viable, colonizing in the bronchial tree and parenchymal tissues, and are essential for health, including immunoregulation, epithelial integrity, and colonization resistance [14]. The main genera have been identified in healthy lungs are *Streptococcus*, *Prevotella*, *Fusobacterium*, *Haemophilus*, and *Veillonella* [15,16]. Compared to the gastrointestinal (GI) tract, which contains 10^11^-10^12^ bacteria per gram of tissue, the microbial population in the lungs of healthy individuals is relatively lower (about 10^3^-10^5^ bacteria per gram of tissue) [17]. It is reported that there are 10–100 bacteria per 1000 human cells in the lung tissue samples [18], and three factors determine its composition: 1) microbial immigration, such as direct mucosal dispersion, bacteria inhalation, and microaspiration; 2) microbial elimination, such as host defence responses, mucociliary clearance, and cough; and 3) regional growth conditions, such as pH, temperature, and nutrient availability [19,20].

It has been shown that both acute and chronic respiratory diseases dramatically alter the lung microbiome composition (Table 1). For instance, patients with idiopathic pulmonary fibrosis (IPF) [35], cystic fibrosis (CF) [36] and bronchiectasis [37] have exhibited an increase in bacterial burden in their lower airways. In addition to changes in the load of bacteria, some specific bacteria are frequently identified in the airways of unhealthy people, such as *Streptococcus*, *Veillonella*, *Neisseria*, and *Haemophilus* (in IPF); S*taphylococcus aureus*, *Pseudomonas aeruginosa*, and *Burkholderia spp*. (in CF); and *Haemophilus*, *P. aeruginosa*, *Prevotella*, and *Veillonella* (in bronchiectasis) [38]. In asthma, bacterial composition and dysbiosis in the lung airway contribute to the severity of the disease and its pathogenesis. Using 16S rRNA-based methods, Huang *et al*. indicated airway dysbiosis in patients with the severe asthmatic condition compared with milder asthma which promotes inflammatory responses by recruiting neutrophils in a Th17-dependent manner. They also found that the lungs of patients with severe asthma were remarkably enriched with *Actinobacteria* and *Klebsiella* [39]. In patients with chronic obstructive pulmonary disease (COPD), the microbiome analysis revealed a reduction in microbial diversity associated with remodelling of the alveolar and bronchiolar tissue, the infiltration of CD4+ *T*-cells, and emphysematous destruction. The COPD patients exhibited an increase in *Actinobacteria* and *Proteobacteria* and reduced *Bacteroidetes* and *Firmicutes*. This alteration in microbiota composition was associated with the infiltration of neutrophils, B-cells, and eosinophils, suggesting the correlation between host immune responses and lung dysbiosis [40]. Furthermore, numerous studies showed that the gut microbiota composition in lung cancer patients is significantly different from healthy control ones, and the respiratory microbiome dysbiosis contributes to inflammation responses and tumorigenesis of lung cancer [41]. A systematic review conducted on 41 studies indicated that pre-existing *Mycobacterium tuberculosis* is associated with the risk of lung cancer [42]. The examination of bronchoalveolar lavage fluid (BALF) samples from 28 patients (20 with lung cancer and 8 at the benign stage) revealed the increase of genera *Megasphaera* and *Veillonella* in patients with lung cancer and TM7 and Firmicutes phyla compared with patients at the benign stage [31].

**Table 1.** Alteration of lung microbiome during respiratory diseases.

Disease	Sample type	Sample size		Microbiome alteration	Ref
CF	BAL		95	**↑***Streptococcus, Staphylococcus, Pseudomonas*	[21]
CF	Sputum		17	**↑***Staphylococcus, Pseudomonas, Achromobacter, Stenotrophomonas*	[22]
CF	BAL		12	**↑***Burkholderia, Streptococcus, Prevotella, Porphyromonas, Haemophilus, Veillonella*	[23]
COPD	Sputum		16	**↑***Pseudomonas, Moraxella, Corynebacterium*	[24]
COPD	Lung tissue		24	**↑***Firmicutes, Lactobacillus*	[18]
COPD	Sputum		8	**↑***Fusobacterium, Bacteroidetes, Actinobacteria, Firmicutes, Streptococcus, ↓Proteobacteria*	[25]
Asthma	Nasopharyngeal		234	**↑***Proteobacteria, Streptococcus*	[26]
Asthma	Bronchoscopy		42	**↑***Neisseria, Haemophilus, Porphyromonas, Fusobacterium*	[27]
Asthma	BAL and endobronchial brushing		58	**↑***Pseudomonas, Lactobacillus, Rickettsia*	[28]
Lung cancer	Saliva		30	**↑***Selenomonas, Veillonella, Capnocytophaga, ↓Streptococcus, Neisseria*	[29]
Lung cancer	Lung tissues and bronchoscopy		42	**↑***Streptococcus, ↓Staphylococcus*	[30]
Lung cancer	BAL		28	**↑***Veillonella, Megasphaera*	[31]
IPF	BAL		55	**↑***Streptococcus, Staphylococcus*	[32]
IPF	BAL		65	**↑***Streptococcus, Veillonella, Haemophilus, Neisseria*	[33]
IPF	Bronchoscopy		35	**↑***Pseudomonas, Streptococcus, Haemophilus, Prevotella, Veillonella*	[34]

CF, cystic fibrosis; BAL, bronchoalveolar lavage; COPD, chronic obstructive pulmonary disease; IPF, idiopathic pulmonary fibrosis.

In addition to the mentioned pathological complications, there is evidence that smoking is another condition that affects the lung microbiota. Smoking and exposure to tobacco could increase the risk of infection with bacteria by increasing the microbial diversity at the lower respiratory tract [43]. Cigarette smoking increases the risk of ARDS in patients following severe trauma. The lung of these patients with smoking status was enriched with pathogenic bacteria, including *Haemophilus*, *Fusobacterium*, *Streptococcus*, *Prevotella*, and *Treponema* [44]. Also, cigarette smoking could alter virus populations in the lungs. Gregory *et al*. indicated that the abundance of bacteriophages infecting *Actinomyces*, *Haemophilus*, *Xanthomonas*, *Rodoferax*, *Aeromonas*, *Prevotella*, and *Capnocytophaga* was increased in smokers, whereas *Morganella* and *Enhydrobacter* bacteriophages were prevailing in non-smokers [45].

## Immune responses against SARS-CoV-2 infection

**Figure 1.**
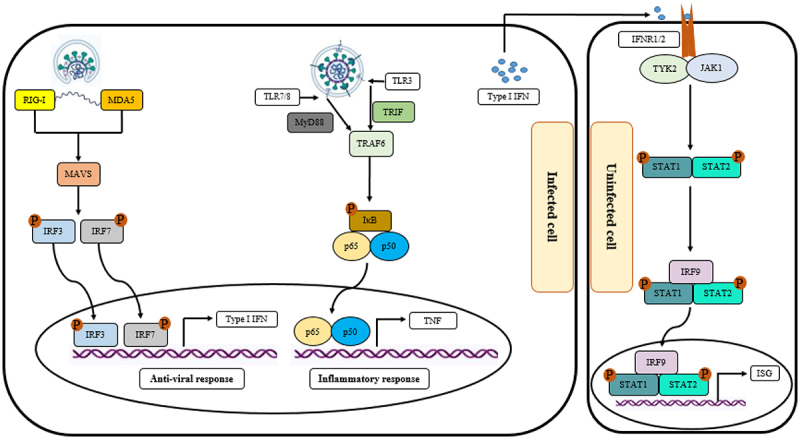
The effect of infection with SARS-CoV-2 on the innate immune response. Following infecting the target cells, the RNA of the virus was recognized by the cytosolic pattern recognition receptors (PRRs), such as the melanoma differentiation-associated protein (MDA5) and the retinoic acid-inducible gene I (RIG-I), which recruit the mitochondrial antiviral signaling protein (MAVS), followed by the phosphorylation and activation of interferon regulatory factor 3 and 7 (IRF3 and IRF7). The phosphorylated form of IRF3 and IRF7 upregulates the expression of type I interferons, as the antiviral response. In addition to the cytosolic receptors, the infection with SARS-CoV-2 is recognized by the endosomal receptors, such as toll-like receptors 3, 7, and 8 (TLR3, TLR7, and TLR8), leading to trigger a cascade to upregulate inflammatory responses. The secreted type I IFNs from the infected cells are recognized through their cognate receptors on the uninfected cells, resulting in the activation of IRF9, STAT1, and STAT2, and, subsequently, upregulation of interferon-stimulated gene (ISG).

The intracellular adherence and tight junctions act as connectors of adjacent cells as well as barriers to regulate paracellular permeability. The impairment of barrier function during diseases increases epithelial permeability and, subsequently, pathogen entry [46]. During infection with viruses, they can bind to their cognate cellular receptors and induce the stimulation of pattern recognition receptors (PRRs) in the epithelial cells, resulting in the secretion of molecules, enzymes, peptides, reactive oxygen species (ROSs), and chemokines with anti-microbial activities. Collectively, the released molecules recruit immune cells and facilitate their communication with each other, contributing to the immune responses that are essential for controlling the infection [47]. In the case of COVID-19, the infection of alveolar type II cells with higher expression of ACE2 receptor through SARS-CoV-2 disrupts the critical functions of these cells, including stabilization of airway epithelial barrier, airway regeneration following injury, and production of pulmonary surfactant [48]. These cells also are involved in the immune responses against pathogens and alveolar damage by producing cytokines to stimulate the recruitment and activation of immune cells, especially macrophages, in defence of the alveolus [49]. Following the binding of the S1 subunit in the viral spike (S) protein, through its receptor-binding domain (RBD), to the receptor, the S protein undergoes protease cleavage by serine protease transmembrane protease serine 2 (TMPRSS2), resulting in uncovering the S2 site and subsequent membrane fusion between the virus and alveolar type II cells, endocytosis of the virus, and release of viral compartments into the cell cytoplasm [50,51]. Neuropilin 1 (NRP1) and Furin also have been identified as co-factors of virus internalization [51,52]. The infected cells release damage-associated molecular patterns (DAMPs) and pathogen-associated molecular patterns (PAMPs), for-example viral RNA, which are recognized by various intracellular PRRs, like RIG-I/MDA5/MAVS/TRAF3/IRF3/IRF7/type I IFNs and TLRs/TRIF/MyD88/TRAF6/p65/p50/TNF pathways (Figure 1). Certainly, targeting these pathways could be beneficial therapeutic strategies in virus-induced diseases. For example, targeting TNF- and IFN-mediated responses during the respiratory syncytial virus infection protects lung cells against the harmful effects of the viral infection [53,54].

The involvement of the host immune system is characterized by the contribution of the innate immune system in the first phase. A typical alveolar immune landscape in the healthy lung is composed mainly of alveolar macrophages located at the air-liquid interface and a few granulocytes, including eosinophils and basophils [55]. Triggering PRR signalling within cells involved in the innate immune responses elevates the levels of pro-inflammatory factors, including tumour necrosis factor (TNF)-α and granulocyte macrophage-colony stimulating factor (GM-CSF) in the plasma of COVID-19 patients as well as interleukin (IL)-1β, IL-6, and IL-8, in their BALF and plasma [56–59]. Additionally, PRR signalling could promote the recruitment and infiltration of neutrophils, monocytes, and *T*-cells to the infection site by upregulating CXCL8, CCL2, and CCL7 [57,60]. Importantly, the signalling of PRR activates the production of anti-viral type I IFNs within plasmacytoid dendritic cells (DCs) [61]. IFNs could stimulate phagocytosis in macrophages and the Janus kinase signal transducer and activator of transcription (JAK-STAT) pathway, which directs the polarization of anti-viral helper *T*-cells [62].

Besides the innate immune system, adaptive immune responses in COVID-19 patients contribute to managing and controlling viral infection. The lysed and damaged epithelium cells in the lung and subepithelial DCs present virus antigens to CD8+ and CD4+ *T*-cells, leading to cytotoxicity activation against virus-infected cells and induction of apoptosis as well as promoting differentiation of CD4+ *T*-cells towards Th1, Th2, Th17, and T follicular helper (FH) [63]. Recently, Pavel *et al*. indicated that the imbalance in Th2/Th1 and Th17/Th1 cytokines could affect the outcomes in patients with SARS-CoV2 infection and is related to their mortality risk [64]. Also, T_FH_ cells help the development of B-cells into plasma cells, promoting the production of virus-specific IgG, IgM, and IgA [63]. Mazzoni *et al*. exhibited that asymptomatic patients showed lower proportions of the viral-specific CD4+ *T*-cells and lower humoral response, suggesting that the frequency of SARS-CoV-2-reactive T-cells is associated with the disease severity. Furthermore, they revealed that asymptomatic individuals show lower multifunctionality of antigen-specific T-cells and lower expression of TIGIT and PD-1 immune-checkpoints compared with symptomatic patients. This indicates that immune responses in symptomatic patients against SARS-CoV-2 can not eliminate the virus rapidly, thus, resulting in repeated activation of immune system cells [65]. In another study, Qin *et al*. found that the population of memory and regulatory T-cells were lessened in COVID-19 patients, whereas naïve T-cell counts were increased compared to healthy ones [66]. Single-cell analysis of T-cells from COVID-19 patients revealed that CD4+ T-cells were activated with high expression of regulatory responses and CD25 as well as suppression of FOXP3 expression in severe COVID-19. These cells showed unique differentiation pathways in the patient’s lungs with both Th1 and Th2 characteristics. In addition, highly activated CD25-expressing CD4+ T-cells facilitated SARS-CoV-2 entry by producing the protease Furin [67].

## Infection with SARS-CoV-2 and microbiome dysbiosis

Since super-infection or co-infection with bacteria partially attributed to the worth outcome and mortality of virus pandemics, including the 1918 H1N1 and 2009 H1N1 influenza, various studies examined this in the infection with COVID-19. The microbiome in humans differs across sex, age, ethnicity, and race, suggesting the unique and specific profiles of the microbial population [68]. The infection of the GI tract by SARS-CoV-2 could disrupt gut microbiota and lead to dysbiosis, GI symptoms, and intestinal inflammation. This imbalance can not be fully restored after even three months of recovery [69]. Also, there is evidence that gut microbiota dysbiosis during infection with SARS-CoV-2 could affect the severity of COVID-19 [70]. It has been shown that gut microbiota could regulate immune responses and modulation of their composition had preventive and therapeutic benefits [71]. For instance, beneficial bacteria *Bifidobacteria* and *Lactobacilli* and butyrate-producing bacteria *Eubacterium rectale* and *Faecalibacterium prausnitzii* were decreased in patients with COVID-19 [72,73], whereas the population of opportunistic bacteria such as *Clostridium hathewayi* and *Clostrium ramosum* were increased and positively correlated with COVID-19 severity [73]. Zuo *et al*. indicated that the faecal samples from patients with COVID-19 were enriched with *Collinsella tanakaei*, *Collinsella aerofaciens*, *Morganella morganii*, and *Streptococcus infantis*, whereas the samples from none-to-low SARS-CoV-2 infectivity displayed a higher abundance of *Lachnospiraceae bacterium 1_1_57FAA*, *Alistipes onderdonkii*, and *Bacteroides stercoris* [74]. Furthermore, there is a negative correlation between the diversity of gut microbiota and COVID-19 severity levels; the diversity decreases with increasing the symptoms of severity in which the “severe” patients show the least diversity [75]. Khan *et al*. demonstrated that the severely COVID-19 patients had elevated levels of IL-21 in comparison with mildly ill and healthy participants, while there were no remarkable differences in the levels of INF-γ and TNF-α among the groups [75]. The investigation of alterations in the gut microbiota of 115 patients and its influence on COVID-19 severity revealed that moderate and severe patients were enriched with *Proteobacteria* compared with mild ones. In contrast, the abundance of butyrate-producing bacteria, including *Lachnospira* and *Roseburia*, and the *Firmicutes/Bacteroidetes* ratio was lower in moderate and severe patients [76]. The produced butyrate, short-chain fatty acids (SCFAs), and propionate with gut microbiota could increase the expression of Tc17 cells and CD8+ cytotoxic T lymphocytes (CTLs). For instance, butyrate could increase granzyme B and IFN-γ expression on CTLs as well as promote the Tc17 cells switch towards cytotoxic phenotypes [77]. In a study, Zhou *et al*. compared the gut microbiota composition and immune responses in moderate COVID-19 patients with and without fever. They found that opportunistic pathogens, including *Saccharomyces cerevisiae* and *Enterococcus faecalis*, were enriched in COVID-19 patients with fever. *S. cerevisiae* was positively associated with diarrhoea symptoms in these patients, whereas *E. faecalis* was positively associated with D-dimer and lactate dehydrogenase (LDH) and negatively correlated with IL-4 and CD8+ *T*-cells. On the other hand, the proportion of species with protective and anti-inflammatory effects, including *Eubacterium ramulus* and *Bacteroides fragilis*, was increased in non-fever patients. Also, there was a negative correlation between enrichment with *E. ramulus* with aspartate aminotransferase (AST), LDH, and IL-6. This study suggested that the gut microbiota contributes to the induction of fever in COVID-19 patients by increasing the pathogenic bacteria in the GI tract, which stimulate the secretion of inflammatory cytokines, including IL-6 [78]. Tao *et al*. found that the changes in the gut microbiota composition is positively correlated with the higher expression of the pro-inflammatory factor IL-18 [79]. Thus, gut microbiota diversity and composition could be act as prognostic biomarkers for COVID-19 severity and immune responses.

## Profiling of the respiratory microbiome in COVID-19 patients

Besides the gut microbiota, infection with SARS-CoV-2 could affect the population and metabolism of respiratory microbiota. The profiling microbiomes of the COVID-19 respiratory tract by Haiminen *et al*. revealed that metabolic pathways are modulated, including a decrease in lipid metabolism (such as sphingolipid metabolism) and glycan metabolism pathways (such as glycan degradation), and an increase in carbohydrate metabolism pathways (such as glycolysis and gluconeogenesis) [80]. It has been shown that sphingosine, as a part of sphingolipids, hinders the interaction of the S subunit of SARS-CoV-2 with ACE2 and reduces the virus infectivity [81]. Also, the changes in glucose metabolism in both innate immune cells and pulmonary epithelial cells contribute to cytokine synthesis and inflammatory responses [82]. Under high glucose conditions, viral replication and cytokine production increase, leading to lung epithelial cell death [83]. Thus, lung microbiome alterations could affect the metabolism in immune cells and lung epithelial cells, affecting the SARS-CoV-2 life cycle. Because bacterial dysbiosis is discussed in detail throughout the article, Table 2. summarizes the dysbiosis of other respiratory microbiota in COVID-19 patients. There is evidence that respiratory viral co-infection in respiratory diseases is 3–68.0% [84]. For instance, Lin et al. stated that viral co-infections in SARS-CoV-2 patients were 3.2% [85]. Furthermore, a metagenomics analysis revealed that in addition to betacoronavirus, COVID-19 samples were associated with other viral co-infections, such as Tombusvirus, Partitivirus, Victorivirus, Totivirus, and Chrysovirus [86].

**Table 2.** Dysbiosis of respiratory microbiota in COVID-19 patients.

Microorganism	Sample type	Sample size	Dysbiosis	Ref
Fungi	BAL	26	- ↑*Candida* spp. - ↑Ascomycota in patients with COVID-19 not colonized with *Candida* spp.	[87]
Fungi	BAS, BAL	90	↑*Candida albicans*	[88]
Fungi	Sputum, EA	99	- ↑*Candida glabrata* - ↑*Candida albicans*	[3]
Virus	Throat swab, Nasal swab, Sputum	23	- ↑*Human alphaherpesvirus 1* - ↑*Rhinovirus B* - ↑ *Human orthopneumovirus*	[89]
Virus	Nasopharyngeal swabs	8	- ↑*Betacoronavirus* - ↓*Alphacoronavirus* - ↓*Cystovirus* - ↑*Macavirus* - ↓*Myovirus*	[90]
Virus	Nasopharyngeal swabs	10	- ↑*Betacoronavirus* - ↓*Siphovirus* - ↓*Alphapapillomavirus* - ↓*Myovirus*	[86]
Archaea	Nasopharyngeal swabs	8	- ↑*Halogeometricum* - ↑*Haloquadratum* - ↑*Natrialba* - ↓*Methanospirillum* - ↓*Methanoregula* - ↓*Methanocaldococcus*	[90]
Archaea	Nasopharyngeal swabs	10	- ↑*Methanosarcina* - ↑*Methanocaldococcus* - ↑*Thermococcus* - ↑*Haloarcula* - ↓*Methanobrevibacter* - ↓*Methanococcus* - ↓*Methanocorpusculum*	[86]

BAL, bronchoalveolar lavage; BAS, bronchial aspirates; EA, endotracheal aspirates.

**Figure 2.**
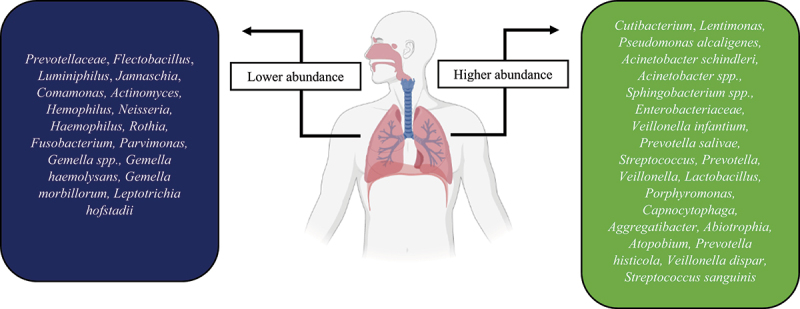
The higher and lower abundance of bacteria in the respiratory tract of COVID-19 patients.

It has been stated that the lung microbiota composition of healthy individuals is mainly enriched with commensal bacteria, including *Tropheryma whipplei*, *Prevotella* spp., *Streptococcus* spp., and *Veillonella* spp., that maintain the immune homoeostasis [91]. The bacterial diversity in COVID-19 patients is controversial; some studies found low microbial diversity [92,93], while others reported a high diversity [94]. These paradoxical results could be owing to the patients’ severity, treatment, disease stage, and differences in sampling location of the respiratory tract. Figure 2 represents the alteration in the respiratory bacteria during infection with COVID-19. To identify the association between upper and lower respiratory tract microbiota and the COVID-19 severity, Lloréns-Rico *et al*. used nasopharyngeal swabs and BAL samples. They explained that the microbiome variation within the upper respiratory tract could be affected by the type of oxygen support (predominantly mechanical ventilation), time in ICU, and treatments (such as antibiotics), while viral load showed a reduced impact [95]. For instance, COVID-19 patients who develop ventilator-associated pneumonia have an impaired innate immune defence that leads to their susceptibility to secondary infection as well as impaired clearance of SARS-CoV-2 infection [96]. Otherwise, there is evidence that the lung microbiome composition between COVID-19 negative and COVID-19 positive patients who developed ventilator-associated pneumonia was similar [97]. Another study revealed that COVID-19 symptomatic patients exhibited low nasopharyngeal bacteria diversity compared with asymptomatic and COVID-19 negative ones. They explained that running sneezing and nose could wash off the nasal microbiota of the symptomatic patients. In the symptomatic patients, nasal microbiota dysbiosis led to high levels of *Cutibacterium* and *Lentimonas*, while reducing the abundance of *Prevotellaceae*, *Flectobacillus*, *Luminiphilus*, *Jannaschia*, and *Comamonas* [98]. Liu *et al*. conducted an investigation to identify metabolome features and nasopharyngeal bacteria of COVID-19 patients. The sera examination of COVID-19 patients revealed that the levels of chlorogenic acid methyl ester (CME), L-proline, and lactic acid were notably reduced compared with COVID-19 negative individuals. Also, the pharynges of these patients were depleted from *Gemella haemolysans*, *Gemella morbillorum*, and *Leptotrichia hofstadii*. In contrast, the population of *Prevotella histicola*, *Veillonella dispar*, and *Streptococcus sanguinis* were increased. They found that the abundance of *L. hofstadii* and *G. haemolysans* were remarkably associated with the serum levels of CME [99]. CME could reduce the expression of inflammatory factors and alleviate the pathological impairment of lung tissue [100]. Gaibani *et al*. characterized the diversity of different bacteria in critically ill COVID-19 patients and healthy subjects using a 16S rRNA profiling on BAL samples. They found that the lung of COVID-19 patients enriched with *Pseudomonas alcaligenes*, *Acinetobacter schindleri*, *Acinetobacter* spp., *Sphingobacterium* spp., and *Enterobacteriaceae*. In contrast, *Veillonella dispar*, *Haemophilus influenzae*, *Granulicatella* spp., *Streptococcus* spp., and *Porphyromonas* spp., characterized in the lung of COVID-19-negative participants [101]. In another study, Merenstein *et al*. indicated the lower abundance of *Actinomyces*, *Hemophilus*, and *Neisseria* in the oropharyngeal of severe COVID-19 patients in comparison with normal ones. They also showed that the lower lymphocyte/neutrophil ratio was associated with the lower microbiome composition and diversity and inversely associated with the disease severity [102]. Iebba *et al*. identified *Veillonella infantium* and *Prevotella salivae* as predominant in patients suffering COVID-19, while *Rothia mucilaginosa* and *Neisseria perflava* were abundant in controls. Furthermore, the levels of cytokines IL-2, IL-5, IL-6, INF-γ, G-CSF, GM-CSF, and TNF-α were augmented in the COVID-19 patients, whereas only IL-12p70 was elevated in control subjects [103]. In a cross-sectional study, Soffritti *et al*. demonstrated that oral microbiome dysbiosis is inversely correlated with the COVID-19 severity. They reported the higher abundance of *Streptococcus*, *Prevotella*, *Veillonella*, *Lactobacillus*, *Porphyromonas*, *Capnocytophaga*, *Aggregatibacter*, *Abiotrophia*, and *Atopobium* in COVID-19 patients, whereas *Haemophilus*, *Rothia*, *Fusobacterium*, *Parvimonas*, and *Gemella* spp. showed lower frequency. Interestingly, the oral fungal and virome in COVID-19 patients were increased in comparison with controls. While *Saccharomyces* spp. and *Candida* were more abundant fungi in control participants, *Nakaseomyces*, *Aspergillus*, and *Malassezia* spp. were high in the COVID-19 patients. Bacteriophages targeted towards *Streptococcus* (phage PH10 and phage EJ-1), *Lactobacillus* (phage phiadh), and *Staphylococcus* (phage ROSA) as well as herpes simplex virus type 1 (HSV-1) were abundant in the COVID-19 patients. Furthermore, a reduction in mucosal sIgA responses was exhibited in more severely COVID-19 patients, suggesting the importance of local immune response in controlling virus infection at the early phase. Among the pro-inflammatory cytokines/chemokines, the levels of IL-6 and IL-17 were meaningfully higher in the oral of COVID-19 patients, without remarkable changes in GM-CSF and TNF-α [104].

There is evidence reporting bacterial, fungal, and viral co-infection with COVID-19. Two meta-analysis studies showed bacterial co-infection in 7% and 14% of hospitalized and critically ill patients, respectively [105,106]. The most commonly reported bacterial co-infection with COVID-19 patients are *Enterobacteriaceae*, *H. influenza*, *and P. aeruginosa* [106,107]. Gaibani *et al*. found that infection with carbapenem-resistant *Acinetobacter baumannii* elevated during COVID-19 [101]. In addition, *Redondoviridae* and *Anelloviridae*, airway commensal DNA viruses, exhibited more colonization frequency in COVID-19 patients and were completely linked to the severity of the disease [102].

## The microbiome and treatments for COVID-19

Due to the undeniable role of microbiota in the COVID-19 disease, management of microbiota to shift its composition towards a healthy state could be effective in controlling SARS-CoV-2 infection and preventing the disease severity. Patients suffering from asymptomatic or mild COVID-19 infections are advised to eat a balanced, healthy, and anti-inflammatory diet, including legumes, fruits, grains, and vegetables. It has been reported that there is a reverse correlation between the amount of fibre intake with inflammatory markers, for example TNF-α, C-reactive protein, IL-6, and IL-18. Furthermore, high-fibre diets are associated with higher adiponectin and lower glycaemia, which have positive anti-inflammatory effects. Also, the anti-inflammatory activities of dietary fibre could support the functions of immunosuppressive and anti-viral drugs [108]. Various studies, including clinical trials, have been directed to investigate the preventive and therapeutic efficacy of microbiota modulation on COVID-19 using probiotics, synbiotics, prebiotics, nutraceuticals, and trace elements. For example, probiotics such as *Paenibacillus* and *Lactobacilli* produce peptides with the binding ability to ACE2, inhibiting the binding of the SARS-CoV-2 to its targeted cells [109,110]. Ceccarelli *et al*. concluded that treatment of patients suffering severe COVID-19 pneumonia with probiotics was correlated with a reduced risk of death [111]. Probiotics also promote anti-viral immune responses by activating toll-like receptor 4 (TLR4) signalling and inducing the secretion of type I and II IFNs [112,113]. Liu *et al*. indicated that orally receiving capsules as faecal microbiota transplantation (FMT) in COVID-19 patients could improve GI symptoms and alter B-cell populations, in which naïve B-cells were decreased and memory and non-switched B-cells were augmented. In discharged patients after FMT administration, the abundance of *Actinobacteria* was increased from 4% to 15%, and the proportion of *Proteobacteria* was reduced from 9.2% to 2.8%. However, they reported abdominal pain and diarrhoea as side effects of FMT administration [114]. In a case report study with two patients, Biliński *et al*. showed that FMT administration to treat concomitant COVID-19 and *C. difficile* infection led to rapid resolution of COVID-19 [115]. A clinical trial is investigating the impact of FMT, as immunomodulatory intervention, on reduced risk of progression of COVID-19 disease with increased inflammatory parameters and cytokine storm (NCT04824222). A meta-analysis study demonstrated that vitamin D deficiency is associated with COVID-19 severity; lower levels in poor prognosis patients [116]. It has been reported that vitamin D could increase the production of butyrate, which improves the gut barrier, exerts anti-inflammatory activities, and promotes anti-viral effects [71]. Table 3. summarizes the clinical trials conducted to evaluate microbiota modulation’s efficacy on COVID-19.

**Table 3.** The effect of nutrients and probiotics in microbiota modulation of COVID-19 under clinical trials.

Intervention/treatment	Participants (n)	Goal	ClinicalTrials.gov Identifier
Probiorinse of *Lactococcus Lactis* W136	23	Reduction of the severity of COVID-19 symptoms	NCT04458519
NSS using *Saccharomyces bourllardii*	80	Reduction of complications in COVID-19 patients and comorbidities	NCT04507867
Combination of *Pediococcus acidilactici* CECT7483, *Lactobacillus plantarum* CECT7484, *L. plantarum* CECT30292, and *L. plantarum* CECT7485	300	How does the combination of the probiotics reduce the risk of progression of COVID-19 to moderate or severe	NCT04517422
*Lactobacillus*	201	Effects on the incidence and severity of COVID-19 in the elderly population living in a nursing home	NCT04756466
NBT-NM108	100	Evaluating the effectiveness and feasibility of NBT-NM108, as a treatment, in modulating gut microbiota of COVID-19.	NCT04540406
Dry extract of polyphenols (tannins) from quebracho and chestnut	124	Evaluating the efficacy of Tannin against COVID-19 infection	NCT04403646
*Lactobacillus Coryniformis* K8	314	Evaluating the protective effect against COVID-19 in healthcare workers	NCT04366180
Probiotic strains with maltodextrin as excipient	41	Improvement of symptoms in COVID-19 patients and reduction of hospitalization days	NCT04390477
*Lactobacillus salivarius* with Vit D and Zinc	60	Effects on immune responses in COVID-19 patients	NCT04937556
*Lactobaciltus rhamnosus* GG	182	Effects on the microbiome of household contacts exposed to COVID-19	NCT04399252
*Lactobacilli* and *Bifidobacteria*	300	Facilitating faster recovery from COVID-19 and enhancing immune responses	NCT04907877

NSS, Nutritional support system.

## Conclusion

Human microbiota can affect immune responses, thus influencing disease progression and prevention. In the case of COVID-19, respiratory microbiota dysbiosis could be associated with underactive and overactive immune responses, which results in various clinical complications. Currently, the respiratory microbiome alteration is effect or cause of the COVID-19 is not clearly distinguished. However, the composition of microbiota differs from person to person and may explain the inter-individual variation in response to COVID-19. Due to the indispensable roles of microbiota in response to COVID-19, the assumption of inexpensive and safe supplementations and nutrients, such as probiotics, can be considered a preventive strategy for COVID-19 negative individuals or an adjunctive for treatment strategies to limit the progression of COVID-19 infection in the suffering patients. Furthermore, personalized diet regimens could direct the therapeutic strategies towards personalized medicine. In the sampling, it should be considered that sampling the lungs for microbial flora sequencing is technically demanding due to the relatively low biomass. In addition, lower respiratory tract sampling by bronchoscopy requires the instrument to be passed through the oral or nasal route, which may lead to the contaminations of samples.

## References

[1] Heymann DL, Shindo N. COVID-19: what is next for public health? Lancet. 2020;395:542–545. 32061313 10.1016/S0140-6736(20)30374-3 PMC7138015

[2] Ferreira-Santos D, Maranhão P, Monteiro-Soares M. Identifying common baseline clinical features of COVID-19: a scoping review. BMJ Open. 2020;10:e041079. 10.1136/bmjopen-2020-041079 PMC7496569 32938604

[3] Chen N, Zhou M, Dong X, et al. Epidemiological and clinical characteristics of 99 cases of 2019 novel coronavirus pneumonia in Wuhan, China: a descriptive study. Lancet. 2020;395:507–513. 32007143 10.1016/S0140-6736(20)30211-7 PMC7135076

[4] Piva S, Filippini M, Turla F, et al. Clinical presentation and initial management critically ill patients with severe acute respiratory syndrome coronavirus 2 (SARS-CoV-2) infection in Brescia, Italy. J Crit Care. 2020;58:29–33. 32330817 10.1016/j.jcrc.2020.04.004 PMC7194649

[5] Quah P, Li A, Phua J. Mortality rates of patients with COVID-19 in the intensive care unit: a systematic review of the emerging literature. Crit Care. 2020;24:1–4. 32498689 10.1186/s13054-020-03006-1 PMC7271132

[6] Gilbert JA, Blaser MJ, Caporaso JG, et al. Current understanding of the human microbiome. Nat Med. 2018;24:392–400. 29634682 10.1038/nm.4517 PMC7043356

[7] Ogunrinola GA, Oyewale JO, Oshamika OO, et al. The human microbiome and its impacts on health. Int J Microbiol 2020. 2020;2020:7. 10.1155/2020/8045646 PMC7306068 32612660

[8] Shang J, Ye G, Shi K, et al. Structural basis of receptor recognition by SARS-CoV-2. Nature. 2020;581:221–224. 32225175 10.1038/s41586-020-2179-y PMC7328981

[9] Segal JP, Mak JWY, Mullish BH, et al. The gut microbiome: an under-recognised contributor to the COVID-19 pandemic? Therap Adv Gastroenterol. 2020;13:1756284820974914. 10.1177/1756284820974914 PMC7692338 33281941

[10] Srinath BS, Shastry RP, Kumar SB. Role of gut-lung microbiome crosstalk in COVID-19. Res Biomed Eng. 2020;38:181–191. 10.1007/s42600-020-00113-4

[11] Gu S, Chen Y, Wu Z, et al. Alterations of the gut microbiota in patients with coronavirus disease 2019 or H1N1 influenza. Clin Infect Dis. 2020;71:2669–2678. 32497191 10.1093/cid/ciaa709 PMC7314193

[12] Rosell A, Monsó E, Soler N, et al. Microbiologic determinants of exacerbation in chronic obstructive pulmonary disease. Arch Intern Med. 2005;165:891–897. 15851640 10.1001/archinte.165.8.891

[13] Thorpe JE, Baughman RP, Frame PT, et al. Bronchoalveolar lavage for diagnosing acute bacterial pneumonia. J Infect Dis. 1987;155:855–861. 3559289 10.1093/infdis/155.5.855

[14] Wang L, Hao K, Yang T, et al. Role of the lung microbiome in the pathogenesis of chronic obstructive pulmonary disease. Chin Med J (Engl). 2017;130:2107. 28741603 10.4103/0366-6999.211452 PMC5586181

[15] Huffnagle GB, Dickson RP, Lukacs NW. The respiratory tract microbiome and lung inflammation: a two-way street. Mucosal Immunol. 2017;10:299–306. 27966551 10.1038/mi.2016.108 PMC5765541

[16] Mathieu E, Escribano-Vazquez U, Descamps D, et al. Paradigms of lung microbiota functions in health and disease, particularly, in asthma. Front Physiol. 2018;9:1168. 30246806 10.3389/fphys.2018.01168 PMC6110890

[17] Yagi K, Huffnagle GB, Lukacs NW, et al. The lung microbiome during health and disease. Int J Mol Sci. 2021;22:10872. 34639212 10.3390/ijms221910872 PMC8509400

[18] Sze MA, Dimitriu PA, Hayashi S, et al. The lung tissue microbiome in chronic obstructive pulmonary disease. Am J Respir Crit Care Med. 2012;185:1073–1080. 22427533 10.1164/rccm.201111-2075OC PMC3359894

[19] Dickson RP, Huffnagle GB, Goldman WE. The lung microbiome: new principles for respiratory bacteriology in health and disease. PLoS Pathog. 2015;11(7):e1004923. 26158874 10.1371/journal.ppat.1004923 PMC4497592

[20] Dickson RP, Martinez FJ, Huffnagle GB. The role of the microbiome in exacerbations of chronic lung diseases. Lancet. 2014;384:691–702. 25152271 10.1016/S0140-6736(14)61136-3 PMC4166502

[21] Frayman KB, Armstrong DS, Carzino R, et al. The lower airway microbiota in early cystic fibrosis lung disease: a longitudinal analysis. Thorax. 2017;72:1104–1112. 28280235 10.1136/thoraxjnl-2016-209279

[22] Feigelman R, Kahlert CR, Baty F, et al. Sputum DNA sequencing in cystic fibrosis: non-invasive access to the lung microbiome and to pathogen details. Microbiome. 2017;5:1–14. 28187782 10.1186/s40168-017-0234-1 PMC5303297

[23] Laguna TA, Wagner BD, Williams CB, et al. Airway microbiota in bronchoalveolar lavage fluid from clinically well infants with cystic fibrosis. PLoS One. 2016;11:e0167649. 27930727 10.1371/journal.pone.0167649 PMC5145204

[24] Millares L, Ferrari R, Gallego M, et al. Bronchial microbiome of severe COPD patients colonised by pseudomonas aeruginosa. Eur J Clin Microbiol Infect Dis. 2014;33:1101–1111. 24449346 10.1007/s10096-013-2044-0 PMC4042013

[25] Lee S-W, Kuan C-S, Ls-H W, et al. Metagenome and metatranscriptome profiling of moderate and severe COPD sputum in Taiwanese Han males. PLoS One. 2016;11:e0159066. 27428540 10.1371/journal.pone.0159066 PMC4948834

[26] Teo SM, Mok D, Pham K, et al. The infant nasopharyngeal microbiome impacts severity of lower respiratory infection and risk of asthma development. Cell Host Microbe. 2015;17:704–715. 25865368 10.1016/j.chom.2015.03.008 PMC4433433

[27] Durack J, Lynch SV, Nariya S, et al. Features of the bronchial bacterial microbiome associated with atopy, asthma, and responsiveness to inhaled corticosteroid treatment. J Allergy Clin Immunol. 2017;140:63–75. 27838347 10.1016/j.jaci.2016.08.055 PMC5502827

[28] Denner DR, Sangwan N, Becker JB, et al. Corticosteroid therapy and airflow obstruction influence the bronchial microbiome, which is distinct from that of bronchoalveolar lavage in asthmatic airways. J Allergy Clin Immunol. 2016;137:1398–1405. 26627545 10.1016/j.jaci.2015.10.017 PMC4860110

[29] Yan X, Yang M, Liu J, et al. Discovery and validation of potential bacterial biomarkers for lung cancer. Am J Cancer Res. 2015;5:3111. 26693063 PMC4656734

[30] Liu H, Tao L, Zhang J, et al. Difference of lower airway microbiome in bilateral protected specimen brush between lung cancer patients with unilateral lobar masses and control subjects. Int J Cancer. 2018;142:769–778. 29023689 10.1002/ijc.31098

[31] Lee SH, Sung JY, Yong D, et al. Characterization of microbiome in bronchoalveolar lavage fluid of patients with lung cancer comparing with benign mass like lesions. Lung Cancer. 2016;102:89–95. 27987594 10.1016/j.lungcan.2016.10.016

[32] Han MK, Zhou Y, Murray S, et al. Lung microbiome and disease progression in idiopathic pulmonary fibrosis: an analysis of the COMET study. Lancet Respir Med. 2014;2:548–556. 24767767 10.1016/S2213-2600(14)70069-4 PMC4142525

[33] Molyneaux PL, Cox MJ, Wells AU, et al. Changes in the respiratory microbiome during acute exacerbations of idiopathic pulmonary fibrosis. Respir Res. 2017;18:1–6. 28143484 10.1186/s12931-017-0511-3 PMC5286769

[34] Molyneaux PL, Maher TM. The role of infection in the pathogenesis of idiopathic pulmonary fibrosis. Eur Respir Rev. 2013;22:376–381. 23997064 10.1183/09059180.00000713 PMC9487348

[35] Molyneaux PL, Cox MJ, Willis-Owen SAG, et al. The role of bacteria in the pathogenesis and progression of idiopathic pulmonary fibrosis. Am J Respir Crit Care Med. 2014;190:906–913. 25184687 10.1164/rccm.201403-0541OC PMC4299577

[36] Muhlebach MS, Zorn BT, Esther CR, et al. Initial acquisition and succession of the cystic fibrosis lung microbiome is associated with disease progression in infants and preschool children. PLoS Pathog. 2018;14:e1006798. 29346420 10.1371/journal.ppat.1006798 PMC5773228

[37] Byun MK, Chang J, Kim HJ, et al. Differences of lung microbiome in patients with clinically stable and exacerbated bronchiectasis. PLoS One. 2017;12:e0183553. 28829833 10.1371/journal.pone.0183553 PMC5567645

[38] Wypych TP, Wickramasinghe LC, Marsland BJ. The influence of the microbiome on respiratory health. Nat Immunol. 2019;20:1279–1290. 31501577 10.1038/s41590-019-0451-9

[39] Huang YJ, Nariya S, Harris JM, et al. The airway microbiome in patients with severe asthma: associations with disease features and severity. J Allergy Clin Immunol. 2015;136:874–884. 26220531 10.1016/j.jaci.2015.05.044 PMC4600429

[40] Sze MA, Dimitriu PA, Suzuki M, et al. Host response to the lung microbiome in chronic obstructive pulmonary disease. Am J Respir Crit Care Med. 2015;192:438–445. 25945594 10.1164/rccm.201502-0223OC PMC4595667

[41] Weinberg F, Dickson RP, Nagrath D, et al. The lung microbiome: a central mediator of host inflammation and Metabolism in lung cancer patients? Cancers (Basel). 2021;13:13. 10.3390/cancers13010013 PMC7792810 33375062

[42] Liang H, Li X, Yu X, et al. Facts and fiction of the relationship between preexisting tuberculosis and lung cancer risk: a systematic review. Int J Cancer. 2009;125:2936–2944. 19521963 10.1002/ijc.24636

[43] Zhang R, Chen L, Cao L, et al. Effects of smoking on the lower respiratory tract microbiome in mice. Respir Res. 2018;19:1–15. 30547792 10.1186/s12931-018-0959-9 PMC6295055

[44] Panzer AR, Lynch SV, Langelier C, et al. Lung microbiota is related to smoking status and to development of acute respiratory distress syndrome in critically ill trauma patients. Am J Respir Crit Care Med. 2018;197:621–631. 29035085 10.1164/rccm.201702-0441OC PMC6005235

[45] Gregory AC, Sullivan MB, Segal LN, et al. Smoking is associated with quantifiable differences in the human lung DNA virome and metabolome. Respir Res. 2018;19:1–13. 30208886 10.1186/s12931-018-0878-9 PMC6136173

[46] Steed E, Balda MS, Matter K. Dynamics and functions of tight junctions. Trends Cell Biol. 2010;20:142–149. 20061152 10.1016/j.tcb.2009.12.002

[47] Invernizzi R, Lloyd CM, Molyneaux PL. Respiratory microbiome and epithelial interactions shape immunity in the lungs. Immunology. 2020;160:171–182. 32196653 10.1111/imm.13195 PMC7218407

[48] Hall MW, Joshi I, Leal L, et al. Immune modulation in COVID-19: strategic considerations for personalized therapeutic intervention. Clin Infect Dis. 2020;74(1):144-148. doi:10.1093/cid/ciaa904. PMC7337699 32604407

[49] Whitsett JA, Alenghat T. Respiratory epithelial cells orchestrate pulmonary innate immunity. Nat Immunol. 2015;16:27–35. 25521682 10.1038/ni.3045 PMC4318521

[50] Tay MZ, Poh CM, Rénia L, et al. The trinity of COVID-19: immunity, inflammation and intervention. Nat Rev Immunol. 2020;20:363–374. 32346093 10.1038/s41577-020-0311-8 PMC7187672

[51] Ou X, Liu Y, Lei X, et al. Characterization of spike glycoprotein of SARS-CoV-2 on virus entry and its immune cross-reactivity with SARS-CoV. Nat Commun. 2020;11:1–12. 32221306 10.1038/s41467-020-15562-9 PMC7100515

[52] Cantuti-Castelvetri L, Ojha R, Pedro LD, et al. Neuropilin-1 facilitates SARS-CoV-2 cell entry and infectivity. Science. 2020;370:856–860. 33082293 10.1126/science.abd2985 PMC7857391

[53] Santos LD, Antunes KH, Muraro SP, et al. TNF-Mediated alveolar macrophage necroptosis drives disease pathogenesis during respiratory syncytial virus infection. Eur Respir J. 2021;57:1–41. 10.1183/13993003.03764-2020 PMC8209485 33303545

[54] Antunes KH, Fachi JL, de Paula R, et al. Microbiota-Derived acetate protects against respiratory syncytial virus infection through a GPR43-type 1 interferon response. Nat Commun. 2019;10:1–17. 31332169 10.1038/s41467-019-11152-6 PMC6646332

[55] Martin TR, Frevert CW. Innate immunity in the lungs. Proc Am Thorac Soc. 2005;2:403–411. 16322590 10.1513/pats.200508-090JS PMC2713330

[56] Zhou Z, Ren L, Zhang LI, et al. Overly exuberant innate immune response to SARS-CoV-2 infection. 2020. 10.2139/ssrn.3551623

[57] Zhou Z, Ren L, Zhang L, et al. Heightened innate immune responses in the respiratory tract of COVID-19 patients. Cell Host Microbe. 2020;27:883–890. 32407669 10.1016/j.chom.2020.04.017 PMC7196896

[58] Liao M, Liu Y, Yuan J, et al. Single-Cell landscape of bronchoalveolar immune cells in patients with COVID-19. Nat Med. 2020;26:842–844. 32398875 10.1038/s41591-020-0901-9

[59] Huang C, Wang Y, Li X, et al. Clinical features of patients infected with 2019 novel coronavirus in Wuhan, China. Lancet. 2020;395:497–506. 31986264 10.1016/S0140-6736(20)30183-5 PMC7159299

[60] Jamilloux Y, Henry T, Belot A, et al. Should we stimulate or suppress immune responses in COVID-19? Cytokine and anti-cytokine interventions. Autoimmun Rev. 2020;19:102567. 32376392 10.1016/j.autrev.2020.102567 PMC7196557

[61] Li G, Fan Y, Lai Y, et al. Coronavirus infections and immune responses. J Med Virol. 2020;92:424–432. 31981224 10.1002/jmv.25685 PMC7166547

[62] Seif F, Khoshmirsafa M, Aazami H, et al. The role of JAK-STAT signaling pathway and its regulators in the fate of T helper cells. Cell Commun Signal. 2017;15:1–13. 28637459 10.1186/s12964-017-0177-y PMC5480189

[63] Azkur AK, Akdis M, Azkur D, et al. Immune response to SARS‐CoV‐2 and mechanisms of immunopathological changes in COVID‐19. Allergy. 2020;75:1564–1581. 32396996 10.1111/all.14364 PMC7272948

[64] Pavel AB, Glickman JW, Michels JR, et al. Th2/th1 cytokine imbalance is associated with higher COVID-19 risk mortality. Front Genet. 2021;12:706902. doi:10.3389/fgene.2021.706902. 34335703 PMC8324177

[65] Mazzoni A, Maggi L, Capone M, et al. Cell‐mediated and humoral adaptive immune responses to SARS‐CoV‐2 are lower in asymptomatic than symptomatic COVID‐19 patients. Eur J Immunol. 2020;50:2013–2024. 33080068 10.1002/eji.202048915

[66] Qin C, Zhou L, Hu Z, et al. Dysregulation of immune response in patients with coronavirus 2019 (COVID-19) in Wuhan, China. Clin Infect Dis. 2020;71:762–768. 32161940 10.1093/cid/ciaa248 PMC7108125

[67] Kalfaoglu B, Almeida-Santos J, Tye CA, et al. T-Cell hyperactivation and paralysis in severe COVID-19 infection revealed by single-cell analysis. Front Immunol. 2020;11:2605. 10.3389/fimmu.2020.589380 PMC7596772 33178221

[68] Consortium HMP. Structure, function and diversity of the healthy human microbiome. Nature. 2012;486:207. 22699609 10.1038/nature11234 PMC3564958

[69] Tian Y, Sun K, Meng T, et al. Gut microbiota may not be fully restored in recovered COVID-19 patients after 3-month recovery. Front Nutr. 2021;8:182. 10.3389/fnut.2021.638825 PMC8155354 34055851

[70] Ferreira C, Viana SD, Reis F. Is gut microbiota dysbiosis a predictor of increased susceptibility to poor outcome of COVID-19 patients? an update. Microorganisms. 2021;9:53. 10.3390/microorganisms9010053 PMC7824665 33379162

[71] Chen J, Vitetta L. Modulation of gut microbiota for the prevention and treatment of COVID-19. J Clin Med. 2021;10:2903. 34209870 10.3390/jcm10132903 PMC8268324

[72] Yeoh YK, Zuo T, Lui G-Y, et al. Gut microbiota composition reflects disease severity and dysfunctional immune responses in patients with COVID-19. Gut. 2021;70:698–706. 33431578 10.1136/gutjnl-2020-323020 PMC7804842

[73] Zuo T, Zhang F, Lui GCY, et al. Alterations in gut microbiota of patients with COVID-19 during time of hospitalization. Gastroenterology. 2020;159:944–955. 32442562 10.1053/j.gastro.2020.05.048 PMC7237927

[74] Zuo T, Liu Q, Zhang F, et al. Depicting SARS-CoV-2 faecal viral activity in association with gut microbiota composition in patients with COVID-19. Gut. 2021;70:276–284. 32690600 10.1136/gutjnl-2020-322294 PMC7385744

[75] Khan M, Mathew BJ, Gupta P, et al. Gut dysbiosis and IL-21 response in patients with severe COVID-19. Microorganisms. 2021;9:1292. 34199203 10.3390/microorganisms9061292 PMC8231954

[76] Moreira-Rosário A, Marques C, Pinheiro H, et al. Gut microbiota diversity and C-Reactive protein are predictors of disease severity in COVID-19 patients. bioRxiv. 2021;12:705020. doi:10.3389/fmicb.2021.705020. PMC8326578 34349747

[77] Luu M, Weigand K, Wedi F, et al. Regulation of the effector function of CD8+ T cells by gut microbiota-derived metabolite butyrate. Sci Rep. 2018;8:1–10. 30258117 10.1038/s41598-018-32860-x PMC6158259

[78] Zhou Y, Shi X, Fu W, et al. Gut microbiota dysbiosis correlates with abnormal immune response in moderate COVID-19 patients with fever. J Inflamm Res. 2021;14:2619. 34168484 10.2147/JIR.S311518 PMC8217908

[79] Tao W, Zhang G, Wang X, et al. Analysis of the intestinal microbiota in COVID-19 patients and its correlation with the inflammatory factor IL-18. Med Microecol. 2020;5:100023. 34173452 10.1016/j.medmic.2020.100023 PMC7832617

[80] Haiminen N, Utro F, Seabolt E, et al. Functional profiling of COVID-19 respiratory tract microbiomes. Sci Rep. 2021;11:1–8. 33742096 10.1038/s41598-021-85750-0 PMC7979704

[81] Törnquist K, Asghar MY, Srinivasan V, et al. Sphingolipids as modulators of SARS-CoV-2 infection. Front Cell Dev Biol. 2021;9:1574. 10.3389/fcell.2021.689854 PMC8245774 34222257

[82] Santos AF, Póvoa P, Paixão P, et al. Changes in glycolytic pathway in SARS-COV 2 infection and their importance in understanding the severity of COVID-19. Front Chem. 2021;9:685196. doi:10.3389/fchem.2021.685196. 34568275 PMC8461303

[83] Codo AC, Davanzo GG, de Brito Monteiro L, et al. Elevated glucose levels favor SARS-CoV-2 infection and monocyte response through a HIF-1α/glycolysis-dependent axis. Cell Metab. 2020;32:437–446. 32697943 10.1016/j.cmet.2020.07.007 PMC7367032

[84] Viciani E, Gaibani P, Castagnetti A, et al. Critically ill patients with COVID-19 show lung fungal dysbiosis with reduced microbial diversity in patients colonized with Candida spp. Int J Infect Dis. 2022;117:233–240. 35150910 10.1016/j.ijid.2022.02.011 PMC8828296

[85] Calderaro A, Buttrini M, Montecchini S, et al. Detection of SARS-CoV-2 and other infectious agents in lower respiratory tract samples belonging to patients admitted to intensive care units of a tertiary-care hospital, located in an epidemic area, during the Italian lockdown. Microorganisms. 2021;9:185. 33467079 10.3390/microorganisms9010185 PMC7830127

[86] Zhong H, Wang Y, Shi Z, et al. Characterization of respiratory microbial dysbiosis in hospitalized COVID-19 patients. Cell Discov. 2021;7:1–14. 33850111 10.1038/s41421-021-00257-2 PMC8043102

[87] Hoque MN, Sarkar M, Hasan M, et al. SARS-CoV-2 infection reduces human nasopharyngeal commensal microbiome with inclusion of pathobionts. Sci Rep. 2021;11:1–17. 34911967 10.1038/s41598-021-03245-4 PMC8674272

[88] Hoque MN, Rahman MS, Ahmed R, et al. Diversity and genomic determinants of the microbiomes associated with COVID-19 and non-COVID respiratory diseases. Gene Rep. 2021;23:101200. 33977168 10.1016/j.genrep.2021.101200 PMC8102076

[89] Hoque MN, Akter S, Mishu ID, et al. Microbial co-infections in COVID-19: associated microbiota and underlying mechanisms of pathogenesis. Microb Pathog. 2021;156:104941. 33962007 10.1016/j.micpath.2021.104941 PMC8095020

[90] Lin X, Gong Z, Xiao Z, et al. Novel coronavirus pneumonia outbreak in 2019: computed tomographic findings in two cases. Korean J Radiol. 2020;21:365–368. 32056397 10.3348/kjr.2020.0078 PMC7039714

[91] Man WH, de Steenhuijsen Piters WAA, Bogaert D. The microbiota of the respiratory tract: gatekeeper to respiratory health. Nat Rev Microbiol. 2017;15:259–270. 28316330 10.1038/nrmicro.2017.14 PMC7097736

[92] Mostafa HH, Fissel JA, Fanelli B, et al. Metagenomic next-generation sequencing of nasopharyngeal specimens collected from confirmed and suspect COVID-19 patients. Mbio. 2020;11: 1- 13. 10.1128/mBio.01969-20 PMC7686804 33219095

[93] Zhang H, Ai J-W, Yang W, et al. Metatranscriptomic characterization of Coronavirus disease 2019 identified a host transcriptional classifier associated with immune signaling. Clin Infect Dis. 2021;73:376–385. 32463434 10.1093/cid/ciaa663 PMC7314197

[94] Han Y, Jia Z, Shi J, et al. The active lung microbiota landscape of COVID-19 patients. medRxiv. 2020;2008.2020.20144014. doi:10.1101/2020.08.20.20144014.

[95] Lloréns-Rico V, Gregory AC, Van Weyenbergh J, et al. Clinical practices underlie COVID-19 patient respiratory microbiome composition and its interactions with the host. Nat Commun. 2021;12:1–12. 34716338 10.1038/s41467-021-26500-8 PMC8556379

[96] Tsitsiklis A, Zha B, Byrne A, et al. Impaired immune signaling and changes in the lung microbiome precede secondary bacterial pneumonia in COVID-19. Res Sq 2021. doi:10.21203/rs.3.rs-380803/v1.

[97] Maes M, Higginson E, Pereira-Dias J, et al. Ventilator-Associated pneumonia in critically ill patients with COVID-19. Crit Care. 2021;25:1–11. 33430915 10.1186/s13054-021-03460-5 PMC7797892

[98] Kolhe R, Sahajpal NS, Vyavahare S, et al. Alteration in nasopharyngeal microbiota profile in aged patients with COVID-19. Diagnostics. 2021;11:1622. 34573964 10.3390/diagnostics11091622 PMC8467337

[99] Liu J, Liu S, Zhang Z, et al. Association between the nasopharyngeal microbiome and metabolome in patients with COVID-19. Synth Syst Biotechnol. 2021;6:135-143. 10.1016/j.synbio.2021.06.002 PMC8200311 34151035

[100] Zhang L, Fan Y, Su H, et al. Chlorogenic acid methyl ester exerts strong anti-inflammatory effects via inhibiting the COX-2/NLRP3/NF-κB pathway. Food Funct. 2018;9:6155–6164. 30379164 10.1039/c8fo01281d

[101] Gaibani P, Viciani E, Bartoletti M, et al. The lower respiratory tract microbiome of critically ill patients with COVID-19. Sci Rep. 2021;11:1–11. 33980943 10.1038/s41598-021-89516-6 PMC8115177

[102] Merenstein C, Liang G, Whiteside SA, et al. Signatures of COVID-19 severity and immune response in the respiratory tract microbiome. mBio. 2021;12:e01777-21. 10.1128/mBio.01777-21 PMC8406335 34399607

[103] Iebba V, Zanotta N, Campisciano G, et al. Profiling of oral microbiota and cytokines in COVID-19 patients. Front Microbiol. 2021; 12:671813. doi:10.3389/fmicb.2021.671813. PMC8361794 34394024

[104] Soffritti I, D’-Accolti M, Fabbri C, et al. Oral microbiome dysbiosis is associated with symptoms severity and local immune/inflammatory response in COVID-19 patients: a cross-sectional study. Front Microbiol. 2021;12:1397. 10.3389/fmicb.2021.687513 PMC8261071 34248910

[105] Langford BJ, So M, Raybardhan S, et al. Bacterial co-infection and secondary infection in patients with COVID-19: a living rapid review and meta-analysis. Clin Microbiol Infect. 2020;26:1622-1629. 10.1016/j.cmi.2020.07.016 PMC7832079 32711058

[106] Lansbury L, Lim B, Baskaran V, et al. Co-Infections in people with COVID-19: a systematic review and meta-analysis. J Infect. 2020;81:266–275. 32473235 10.1016/j.jinf.2020.05.046 PMC7255350

[107] Zhu X, Ge Y, Wu T, et al. Co-Infection with respiratory pathogens among COVID-2019 cases. Virus Res. 2020;285:198005. 32408156 10.1016/j.virusres.2020.198005 PMC7213959

[108] Conte L, Toraldo DM. Targeting the gut–lung microbiota axis by means of a high-fibre diet and probiotics may have anti-inflammatory effects in COVID-19 infection. Ther Adv Respir Dis. 2020;14:1753466620937170. 32600125 10.1177/1753466620937170 PMC7328354

[109] Li J, Zhao J, Wang X, et al. Novel angiotensin-converting enzyme-inhibitory peptides from fermented bovine milk started by Lactobacillus helveticus KLDS. 31 and Lactobacillus casei KLDS. 105: purification, identification, and interaction mechanisms. Front Microbiol. 2019;10:2643. 31849852 10.3389/fmicb.2019.02643 PMC6892751

[110] Minato T, Nirasawa S, Sato T, et al. B38-CAP is a bacteria-derived ACE2-like enzyme that suppresses hypertension and cardiac dysfunction. Nat Commun. 2020;11:1–12. 32103002 10.1038/s41467-020-14867-z PMC7044196

[111] Ceccarelli G, Borrazzo C, Pinacchio C, et al. Oral bacteriotherapy in patients with COVID-19: a retrospective cohort study. Front Nutr. 2021;7:341. 10.3389/fnut.2020.613928 PMC7829198 33505983

[112] Kumova OK, Fike AJ, Thayer JL, et al. Lung transcriptional unresponsiveness and loss of early influenza virus control in infected neonates is prevented by intranasal Lactobacillus rhamnosus GG. PLoS Pathog. 2019;15:e1008072. 31603951 10.1371/journal.ppat.1008072 PMC6808501

[113] Eguchi K, Fujitani N, Nakagawa H, et al. Prevention of respiratory syncytial virus infection with probiotic lactic acid bacterium Lactobacillus gasseri SBT2055. Sci Rep. 2019;9:1–11. 30886158 10.1038/s41598-019-39602-7 PMC6423325

[114] Liu F, Ye S, Zhu X, et al. Gastrointestinal disturbance and effect of fecal microbiota transplantation in discharged COVID-19 patients. J Med Case Rep. 2021;15:1–9. 33557941 10.1186/s13256-020-02583-7 PMC7868905

[115] Biliński J, Winter K, Jasiński M, et al. Rapid resolution of COVID-19 after faecal microbiota transplantation. Gut. 2022;71:230–232. 34230217 10.1136/gutjnl-2021-325010

[116] Munshi R, Hussein MH, Toraih EA, et al. Vitamin D insufficiency as a potential culprit in critical COVID‐19 patients. J Med Virol. 2021;93:733–740. 32716073 10.1002/jmv.26360

